# The MYST-Containing Protein Chameau Is Required for Proper Sensory Organ Specification during *Drosophila* Thorax Morphogenesis

**DOI:** 10.1371/journal.pone.0032882

**Published:** 2012-03-06

**Authors:** Matthieu Hainaut, Thierry Sagnier, Hélène Berenger, Jacques Pradel, Yacine Graba, Benoit Miotto

**Affiliations:** Institut de Biologie du Développement de Marseille-Luminy, CNRS UMR6216/Université de la Méditerranée, Marseille, France; Ludwig-Maximilians-Universität München, Germany

## Abstract

The adult thorax of *Drosophila melanogaster* is covered by a stereotyped pattern of mechanosensory bristles called macrochaetes. Here, we report that the MYST containing protein Chameau (Chm) contributes to the establishment of this pattern in the most dorsal part of the thorax. Chm mutant pupae present extra-dorsocentral (DC) and scutellar (SC) macrochaetes, but a normal number of the other macrochaetes. We provide evidences that *chm* restricts the singling out of sensory organ precursors from proneural clusters and genetically interacts with transcriptional regulators involved in the regulation of achaete and scute in the DC and SC proneural cluster. This function of *chm* likely relies on chromatin structure regulation since a protein with a mutation in the conserved catalytic site fails to rescue the formation of supernumerary DC and SC bristles in *chm* mutant flies. This is further supported by the finding that mutations in genes encoding chromatin modifiers and remodeling factors, including Polycomb group (PcG) and Trithorax group (TrxG) members, dominantly modulate the penetrance of *chm* extra bristle phenotype. These data support a critical role for chromatin structure modulation in the establishment of the stereotyped sensory bristle pattern in the fly thorax.

## Introduction

Twenty-six large sensory bristles (or macrochaetes) are arranged in a stereotyped pattern on the dorsal thorax of *Drosophila melanogaster*. The regulation of macrochaete number and position has been widely studied as a paradigm to decipher genetic and molecular mechanisms involved in epithelium regionalization and differentiation (reviewed in [1]–[7]). The macrochaete is composed of five cells issued from the asymmetric division of a unique progenitor, the sensory organ precursor or SOP [8]. Each SOP is selected during larval development from a proneural territory comprising 10 to 30 cells of the wing imaginal disc epithelium that are provided with the potential to develop a neural fate by bHLH (basic helix-loop-helix) transcriptional activators encoded by proneural genes of the *achaete-scute* (*ac*-*sc*) complex [9]–[11]. Proneural genes are controlled by the products of so-called prepattern genes, whose regulatory activities are integrated by multiple, independent cis-regulatory elements scattered in the *ac*-*sc* locus [3], [4]. Within the proneural cluster, SOP singling out results from coordinated processes of Notch-mediated lateral inhibition and autoregulatory activation loop, leading to increased accumulation of Ac-Sc factors in the SOP. Conversely, different classes of transcriptional repressors that prevent *ac-sc* expression promote the epidermal fate of non-SOP cells [12]–[19]. Once selected, SOPs enter differentiation and neuronal identity programs under combinatorial controls involving prepattern, proneural and tissue-specific gene products (reviewed in [4], [7]).

The *pannier* (*pnr*) gene encodes a transcription factor of the GATA family, which is a key morphogenetic factor for dorsal identity specification in flies (reviewed in [3]). Pnr noteworthily promotes the development of dorsocentral (DC) and scutellar (SC) sensory organs in the mesothorax, which are part of the stereotyped bristle pattern that decorates the adult thorax [20], [21]. The determination of DC bristles is widely studied in particular because an enhancer specifically recapitulating *ac-sc* expression in the DC proneural cluster has been cloned and dissected. For instance, Wingless (Wg) signaling provides a permissive environment for *ac*-*sc* expression [22]. Several transcription factors including Pnr, Chip (Chi), dLMO, Daughterless and possibly Ac (see however ref. 4), are required for the activation of the DC enhancer. It has been proposed that these factors function within a multimeric complex where Chi acts as a bridge between Pnr and Ac-Daughterless heterodimer to allow enhancer/promoter communication and transcriptional initiation [23]–[25]. A Iswi/Toutatis chromatin complex is also recruited to facilitate enhancer/promoter communication and proneural activity [26]. Several directly interacting co-repressors down-regulate Pnr-mediated transcriptional activation of *ac-sc* in the DC proneural cluster, such as U-shaped (Ush) [27], dCtBP [28] and the member of the Brahma (Brm) chromatin remodeling complex Osa [29]. The molecular regulation of SC bristles determination, on the other hand, is poorly understood. In particular, the regulatory elements controlling *ac-sc* expression in the SC proneural cluster are yet to be described. Nevertheless, mutations of several, but not all of the factors involved in DC determination also alter the pattern of SC bristles: Pnr, Ush and the Brm complex, are involved in DC and SC determination.

The MYST containing protein encoded by *chameau* (*chm*) has been initially identified as an epigenetic regulator of transcription involved in distinct mechanisms: modulation of Hox regulatory functions of Polycomb group (PcG) [30] and Trithorax group (TrxG) proteins (unpublished); control of JNK/AP-1 signaling during metamorphosis and thoracic closure [31]; regulation of the assembly and recruitment of the replication machinery at the vicinity of a replication origin [32]. In addition, *chm* has also been associated to the dendritic patterning of sensory neurons during embryogenesis [33], to the formation of the dorso-ventral boundary in wing imaginal discs [34] and to odor-guided behavior in adult flies [35]. Here, we report on the function of Chm in the control of macrochaete patterning in the *Drosophila melanogaster* thorax.

## Results

### *chm* mutants display extra bristle phenotype

**Figure 1:**
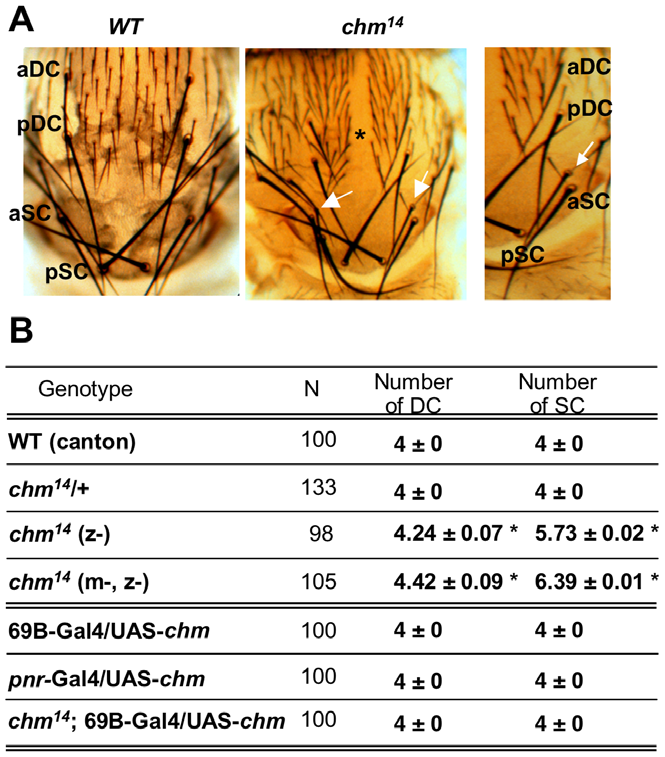
*chm* is required for the development of proper number of aDC and aSC macrochaetes. (A) Cuticle preparation of WT and *chm* pharate adult thoraces. White arrows point towards supernumerary aSC bristles with their proper socket cells, and the asterix to the thoracic cleft induced by *chm* mutation. aDC: anterior DC, pDC: posterior DC, aSC: anterior SC, pSC: posterior SC. (B) Summary of the genetic characterization of *chm* mutant bristle phenotype, and phenotypic rescue by UAS-*chm*. Bristle scoring were compared using Student's *t* test: *, statistically different with regards to WT flies, *P*<0.05. N, number of thoraces examined. z-, zygotic mutant. m-, z-, maternal and zygotic mutant, s.e.m of 3 independent experiment is indicated.

Homozygous *chm^14^* and *chm^221^* mutants carry deletions spanning part of the *chm* coding sequence and are zygotically null [30]. These animals develop up to the pharate adult stage but fail to emerge from the pupal cage. They present morphological defects of external cuticle including a thoracic cleft and an increased number of mechanosensory organs (Fig. 1A; [31]). Supernumerary bristles in the *chm* mutant are only observed for SC and DC machrochaetes. While heterozygous animals, as wild-type flies, display four SC and four DC macrochaetes, *chm* pharate adults exhibit supernumerary anterior SC (aSC, an average of 5.73 per animal) and anterior DC (aDC, 4.24 per animal) (Fig. 1B). Compared to the normal, the extra bristles are thinner and shorter, but contain socket cells of apparently normal morphology (Fig. 1A). Depleting the maternal contribution of *chm* slightly aggravates the penetrance of this phenotype. Thus, 11% of *chm* zygotic mutants present extra aDC and 88% extra aSC, compared to 30% of *chm* mutants deprived of both maternal and zygotic contributions that exhibit supernumerary aDC and 100% supernumerary aSC, with an average of 4.42 aDC and 6.39 aSC per animal (Fig. 1B). However, zygotic or maternal plus zygotic loss of Chm results in qualitatively similar sensory bristle phenotypes, which only consist in the appearance of supernumerary DC and SC macrochaetes.

Because *chm* loss-of-function animals present extra aSC and aDC bristles, we investigated the impact of a gain-of-function in the wing imaginal disc on mechanosensory organ formation. *UAS-chm* transgenic flies were crossed with various Gal4 drivers (heat-shock-Gal4 with a heat pulse during the third larval stage, 69B-Gal4, *arm*-Gal4; *pnr*-Gal4). In all combinations we could follow expression of the Myc-tagged Chm protein (data not shown), but failed to notice any alteration of the WT phenotype (Fig. 1B), indicating that an excess of Chm does not affect thorax development. Constitutive *chm* overexpression in a *chm* mutant background perfectly rescues thoracic defects (Fig. 1B [31]), establishing that the UAS-Chm transgene is functional and that the phenotypes originate from the lack of *chm* activity. Driving *chm* under the control of *pnr*-Gal4 in the most dorsal part of wing discs similarly allows normal thorax morphogenesis and proper number of SC and DC bristles to be specified. Thus, Chm plays a crucial role during thorax closure and macrochaete development.

We previously reported that *chm* controls the activity of the JNK signaling pathway in the proximal part of the wing imaginal disc epithelium during thoracic closure [31]. In order to check whether Chm also controls JNK signalling during bristle specification, we performed genetic interaction assays between *chm* and genes encoding key factors of the JNK pathway. Reducing the gene dosage of *kayak*, *hemipterous* or *jra/djun*, which aggravates *chm* thorax closure defect [31], does not significantly modify the frequency and number of supernumerary SC and DC macrochaetes present in *chm* mutant pharate adults (Table 1). This result strongly suggests that the control of sensory bristle formation by Chm occurs in a manner that does not depend on its ability to modulate JNK signaling.

**Table 1:** *kay*, *hep* or *jra/Djun* do not dominantly interact with *chm* during DC and SC macrochaete development.

Genotype	N	Number of DC	Number of SC
***chm^14^***	98	**4.24±0.07**	**5.73±0.02**
***chm^14^*** **; ** ***kay^1^*** **/+**	33	**4.14±0.05** ns	**5.72±0.08** ns
***hep^1^*** **/+; ** ***chm^14^***	60	**4.12±0.04** ns	**5.58±0.12** ns
***hep^75^*** **/+; ** ***chm^14^***	32#	**4.06** ns	**5.58** ns
***chm^14^*** **/** ***chm^14^*** **, ** ***jra^IA109^***	87	**4.14±0.07** ns	**5.86±0.03** ns

Bristle scoring was compared using Student's *t* test. ns, not statistically different with regards to *chm* mutant flies. N, number of thoraces examined.

# , total number of individuals scored for this genotype by pulling data from different independent crosses.

DC: dorsocentral bristles, SC: scutellar bristles.

### *chm* controls SOP specification

**Figure 2:**
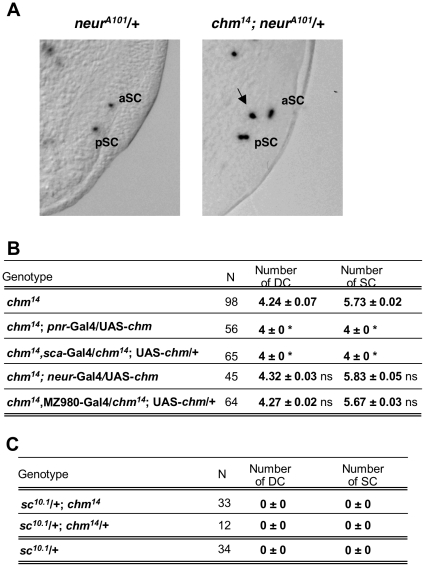
*chm* controls SOP singling out from proneural clusters. (A) Scutellar parts of the posterior notum of wing imaginal discs from a WT late third instar larva (left panel) and from a *chm* mutant white pupa (right panel) stained for SOPs using the *neur^A101^* LacZ enhancer trap strain. The arrow indicates an ectopic SOP singling out in the chm mutant disc. (B) Phenotypic rescue of *chm* mutant supernumerary bristle phenotype upon providing Chm back in *pnr*, *sca* and *neur* expression domains, and in the proximal part of the notum (MZ980). Bristle scoring was compared using Student's *t* test: *, statistically different with regards to *chm* mutant flies, *P*<0.05; ns, not statistically different. N, number of thoraces examined. s.e.m of 3 independent experiment is indicated (C) Extra-SOPs in *chm* mutants required ac/sc transcription. N, number of thoraces examined.

To approach the role of *chm* in SOP determination, we first used a LacZ enhancer trap in *neuralized* (*neur*), *neur^A101^*, which labels SOPs and their progeny [36]. As *chm* mutation more frequently induces the formation of supernumerary SC macrochaetes, we focused on SOPs emerging from the SC proneural cluster in the notum of imaginal wing discs. While two SOPs (one aSC and one posterior SC) are generated in the SC cluster of wild type wing discs, the LacZ pattern of *neur^A101^* reveals three SOPs in *chm* mutant discs (Fig. 2A). The ectopic SOP localizes in the presumptive region of the wing imaginal disc where the ectopic bristle will form. The extra SOP is determined later than normal SOPs, at a time where aSC and posterior SC have already completed the first asymmetric division, and emerges at a distance of the normal aSC with unlabelled cells in between. Therefore, additional SC bristles in *chm* mutant do not result from a defect in the bristle differentiation lineage and/or in SOP asymmetric division.

To assess whether Chm could act early in proneural clusters to influence SOP singling out, we performed phenotypic rescue experiments using various drivers: *scabrous*(*sca*)-Gal4, *pnr*-Gal4, *neur*-Gal4 and MZ980-Gal4. Expression of UAS-*chm* under *sca*-Gal4 in DC and SC proneural clusters or under *pnr*-Gal4 in the dorsal part of a prospective thoracic region that includes DC and SC proneural clusters, perfectly rescues *chm* supernumerary bristle phenotype. As a control, using MZ980-Gal4, that drives expression specifically in a wing disc proximal region distinct from DC and SC clusters and later along the prospective junction of the contralateral discs [37], perfectly rescues the thoracic cleft of *chm* mutants [31] but not the extra bristle phenotype. Interestingly, UAS-*chm* driven by *neur*-Gal4, which promotes expression later than *sca*-Gal4, when SOPs have already been singled out, does not allow supernumerary bristle rescue (Fig. 2B). Therefore, *chm* function in DC and SC proneural clusters is required early in the process of SOP specification prior to SOP specification.

*Df(1)^sc10-1^*/Y living males are null for *ac*-*sc* and display a complete absence of thoracic sensory organs [38]. *chm* mutation does not restore SC and DC bristle development in the *Df(1)^sc10-1^*/Y genetic background (Fig. 2C). This genetic epistatic relationship indicates that *chm* cannot provide proneural activity in the absence of *ac*-*sc*. Thus, *chm* may act upstream of *ac-sc* to repress or restrict their expression in the proneural cluster. Alternatively, Chm may reduce Ac and Sc proneural activities in the proneural cluster to prevent excess of SOP specification.

### *chm* genetically interacts with the GATA factor Pnr encoding gene during SOP specification

Phenotypes observed in gain-of-function mutants of *pnr* as well as in combinations of hypomorphic loss-of-function *pnr* alleles indicate that Pnr is required for thoracic closure and for DC and SC bristle specification as well [20], [21]. The resemblance of *chm* loss-of-function phenotypes and *pnr* gain-of-function phenotypes prompted us to test for genetic interactions between null alleles of *chm* and well-characterized mutations in *pnr*: the *pnr^D1^* allele that produces a truncated protein no longer able to bind the Ush protein, known to act as an antagonist of Pnr activity in specific contexts [27], [39]; the *pnr^VX1^* and *pnr^VX6^* loss-of-function hypomorphic and *pnr^VX2^* null alleles [21].

Scoring DC macrochaetes, we observed that *pnr^VX^* mutations dominantly suppress and that *pnr^D1^*dominantly enhances *chm* extra DC bristle phenotype (Table 2). Of note, *pnr^D1^* heterozygous flies present ectopic DC bristles, which is enhanced further upon *chm* mutation, indicating that the two genes have opposite functions in macrochaete specification originating from the DC proneural cluster.

**Table 2:** *chm* genetically interacts with *pnr* during DC and SC macrochaete development.

Genotype	N	Number of DC	Number of SC
**WT (canton)**	100	**4±0** *	**4±0** *
***chm^14^***	98	**4.24±0.07**	**5.73±0.02**
***chm^14^; pnr^D1^*** **/** ***+***	54	**7.48±0.07** *	**4.39±0.03** *
***chm^14^; pnr^VX1^*** **/** ***+***	57	**4±0** *	**4.19±0.12** *
***chm^14^; pnr^VX2^*** **/** ***+***	55	**4±0** *	**4.78±0.05** *
***chm^14^; pnr^VX6^*** **/** ***+***	55	**4±0** *	**4.76±0.03** *
***pnr^D1^*** **/** ***+***	75	**6.11±0.03**	**4.21±0.02**
***pnr^VX1^*** **/** ***+***	57	**4±0**	**4±0**
***pnr^VX2^*** **/** ***+***	50	**4.01±0.01**	**4±0**
***pnr^VX6^*** **/** ***+***	50	**4±0**	**4.02±0.03**

Bristle scoring was compared using Student's *t* test.

* , statistically different compared to *chm* null flies, *P*<0.05.

N, number of thoraces examined. DC: dorsocentral bristles, SC: scutellar bristles.

Scoring SC macrochaetes, we observed that *pnr^VX^* mutants dominantly suppress the *chm* phenotype (Table 2), again consistent with opposite roles for the two genes. Surprisingly, *pnr^D1^* was found to also suppress *chm* extra SC bristle phenotype, which was not expected from above-mentioned genetic interactions during DC macrochaete formation. This suggests that unlike for DC bristle specification, *pnr^D1^* does not behave as a gain-of-function allele for macrochaete singling out from the SC proneural cluster. This also highlights that despite sharing common regulators, SOP specification likely also relies on mechanisms specific to DC and SC proneural clusters.

### *chm* genetically interacts with genes encoding transcriptional coregulators of Pnr

**Figure 3:**
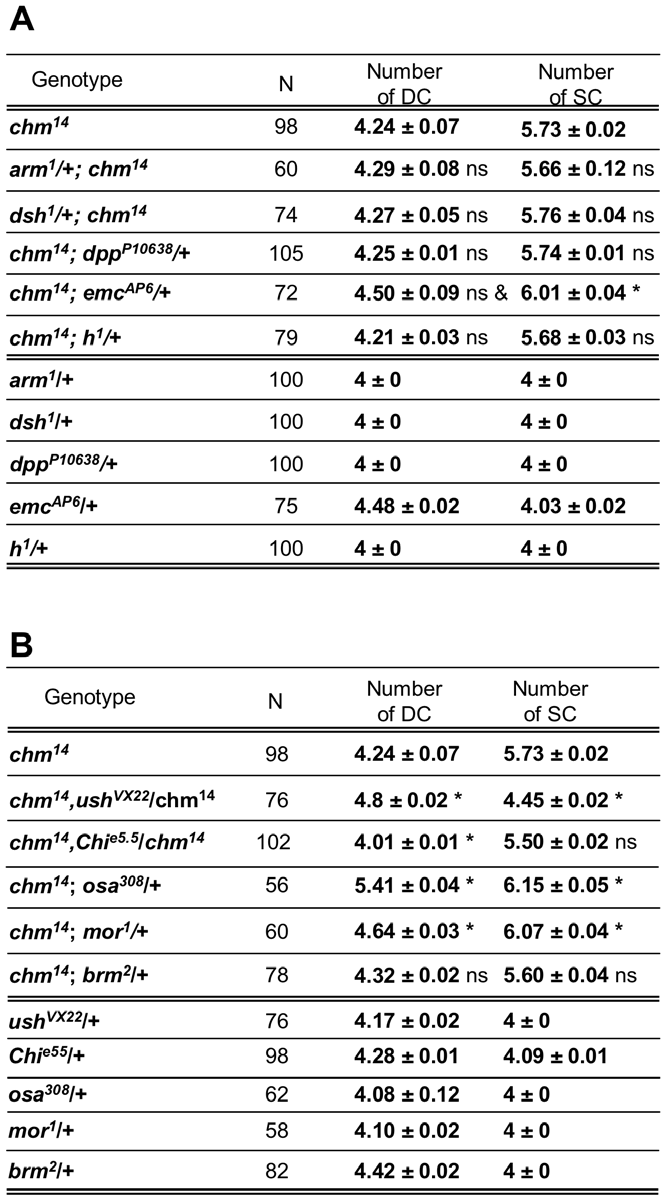
*chm* genetically interacts with transcriptional cofactors of Pnr. (A) Summary of genetic interactions between *chm* and genes involved in the pre-patterning of the thorax. Bristle scoring were compared using Student's *t* test: *, statistically different with regards to *chm* mutant flies, *P*<0.05; ns, not statistically different. N, number of thoraces examined. &, non significant as *emc^AP6^* heterozygotes have an average of 4.48 DC. (B) Summary of genetic interactions between *chm* and genes involved in the regulation of Pnr transcriptional activity. Bristle scoring were compared using Student's *t* test: *, statistically different with regards to *chm* mutant flies, *P*<0.05; ns, not statistically different. N, number of thoraces examined.

Several transcription factors and signaling pathways together with Pnr control the location of DC and SC proneural clusters in the epithelium and thus, the cells where *ac-sc* will be activated. Pnr cooperates, among others, with Wingless (Wg) and Decapentaplegic (Dpp) signaling pathways in the patterning of the dorsal thorax. This prompted us to test for interactions between alleles of the *dpp* and *wg* pathways and *chm*. Scoring SC and DC macrochaetes, we did not observe a qualitative alteration of the *chm* phenotype by mutation of *dpp* and *wg* signaling effectors *armadillo* (*arm*) and *disheveled* (*dsh*) (Fig. 3A). These results suggest that *chm* may not regulate the function of Pnr as a pre-pattern gene, consistent with the rescue experiment with the *sca*-Gal4 driver.

On the other hand Pnr activates the expression of *ac* and *sc* in the DC and SC proneural clusters [22], [23]. Extensive molecular and genetic studies have identified multiple partners of Pnr in the regulation of DC and SC bristle specification. In particular, Chi, Dlmo, the bHLH proteins Ac/Sc and Daughterless may participate in the transcriptional activation of proneural genes in the DC cluster. Several additional factors are known to repress Pnr-mediated transactivation in the DC cluster, including Ush and TrxG group members of the Brm complex. Accordingly, mutations in the encoding genes result, as for *chm* and *pnr*, in the formation of extra-DC and/or SC macrochaetes or, on the contrary, the lack of DC and/or SC [23]–[25]. This prompted us to test for interactions between these genes and *chm*. Significant genetic interactions were observed with *ush*, *Chi*, *osa* and *brm* (Fig. 3B). The mutation of one copy of *Chi*, which cooperates with *pnr* in *ac-sc* transcriptional activation, suppresses the extra DC bristle phenotype of *chm* mutants without affecting the extra SC bristle phenotype. Thus, *Chi* antagonizes *chm* activity in a context-dependent manner, for DC macrochaete specification. The null allele of *ush*, *ush^VX22^*, dominantly enhances the penetrance of extra DC and dominantly suppresses that of extra SC bristles seen in *chm* mutants. Note that heterozygosis for *ush^VX22^* or for *pnr^D1^* (Table 1) similarly modifies *chm* bristle phenotypes. Lastly, mutating one copy of one of the TrxG genes *osa* and *moira (mor)* enhances both DC and SC *chm* phenotypes (Fig. 3B), in agreement with their products being part of the Brm complex. Surprisingly, no genetic interaction was detected with *brm* that encodes the third TrxG member of the Brm complex (Fig. 3B), suggesting that in the absence of Chm one dose of the *brm* gene is sufficient to maintain Brm complex function. Together these results indicate that *chm* genetically interacts with *pnr* and several of its co-factors in DC and SC specification. In addition, the context-specific effects of several genetic combinations suggest that the control of *ac-sc* by these factors differ in its requirement for chromatin remodeling, histone modifications and co-factors in the DC and SC proneural clusters. As a corollary, *chm* does not repress SOP emergence through a unique and shared mechanism in the SC and DC proneural clusters.

This context specific effect was further supported by looking at genetic interactions with two additional genes involved in the pre-patterning of the thorax. Extra macrochaete (Emc), a helix-loop-helix factor lacking the basic DNA binding domain, sequesters Ac and Sc in heterodimers unable of binding to DNA [40]–[42]. The mutation of one copy of *emc* dominantly enhances the *chm* supernumerary SC bristle phenotype without altering the extra DC bristle phenotype (Fig. 3A). Thus, *emc* synergizes with *chm* activity in a context-dependent manner for SC macrochaete specification. Finally, *hairy* shows extensive similarity with transcription factor N-myc and is also involved in bristle patterning [43]. The mutation of one copy of *hairy* does not modify the *chm* supernumerary SC or DC phenotype (Fig. 3A).

### *chm* bristle phenotype is not altered by mutants of the lateral inhibition process

Lateral inhibition is important to restrict the neural fate to the SOP in the proneural cluster. This is achieved by cell-cell communication where signals emanating from the SOP will repress the neural fate of neighboring cells. An important player in this process is the Notch signalization [44]. We thus tested whether mutations of *Enhancer of split* (*E(spl)*), *Hairless* (*H*) and *Notch* (*N*), which mediate Notch pathway activity, will genetically interact with *chm* (Table 3). We found no genetic interaction between *chm* and these mutants. These data are consistent with a genome-wide RNAi screen in *Drosophila* cell culture aiming at the identification of genes that control Notch-dependent transcription. In the screen, depletion of *chm* does not significantly affect Notch-dependent transcription [45]. Thus, extra-SOP present in *chm* mutants may not result from a defect in lateral inhibition.

**Table 3:** *chm* does not genetically interacts with mutants of the Notch pathway during DC and SC macrochaete development.

Genotype	N	Number of DC	Number of SC
**WT (canton)**	100	**4±0** *	**4±0** *
***chm^14^***	98	**4.24±0.07**	**5.73±0.02**
***E(spl)^1^; chm^14^***	136	**4.11±0.07 **ns	**5.72±0.03 **ns
***E(spl)^R1^; chm^14^***	89	**4.17±0.03 **ns	**5.64±0.03 **ns
***E(spl)^R1^/E(spl)^1^, chm^14^*** **/** ***+***	47	**5.80±0.12** *	**5.46±0.07 **ns
***N^55e1^/+; chm^14^***	45	**4.42±0.03 **ns	**5.74±0.08 **ns
***chm^14^; H^1^*** **/** ***+***	58	**3.32±0.23** *	**5.46±0.08 **ns
***E(spl)^1^*** **/** ***+***	120	**4±0**	**4±0**
***E(spl)^R1^*** **/** ***+***	119	**4.88±0.05**	**4.28±0.03**
***E(spl)^R1^*** **/** ***E(spl)^1^***	54	**5.45±0.05**	**5.22±0.04**
***N^55e1^*** **/** ***+***	34	**4.09±0.02**	**4±0**
***H^1^*** **/** ***+***	56	**3.10±0.12**	**4±0**

Bristle scoring was compared using Student's *t* test.

* , statistically different compared to *chm* null flies, *P*<0.05.

ns, not statistically different with regards to *chm* mutant flies. N, number of thoraces examined. DC: dorsocentral bristles, SC: scutellar bristles.

### Chm epigenetically represses SOP formation

**Figure 4:**
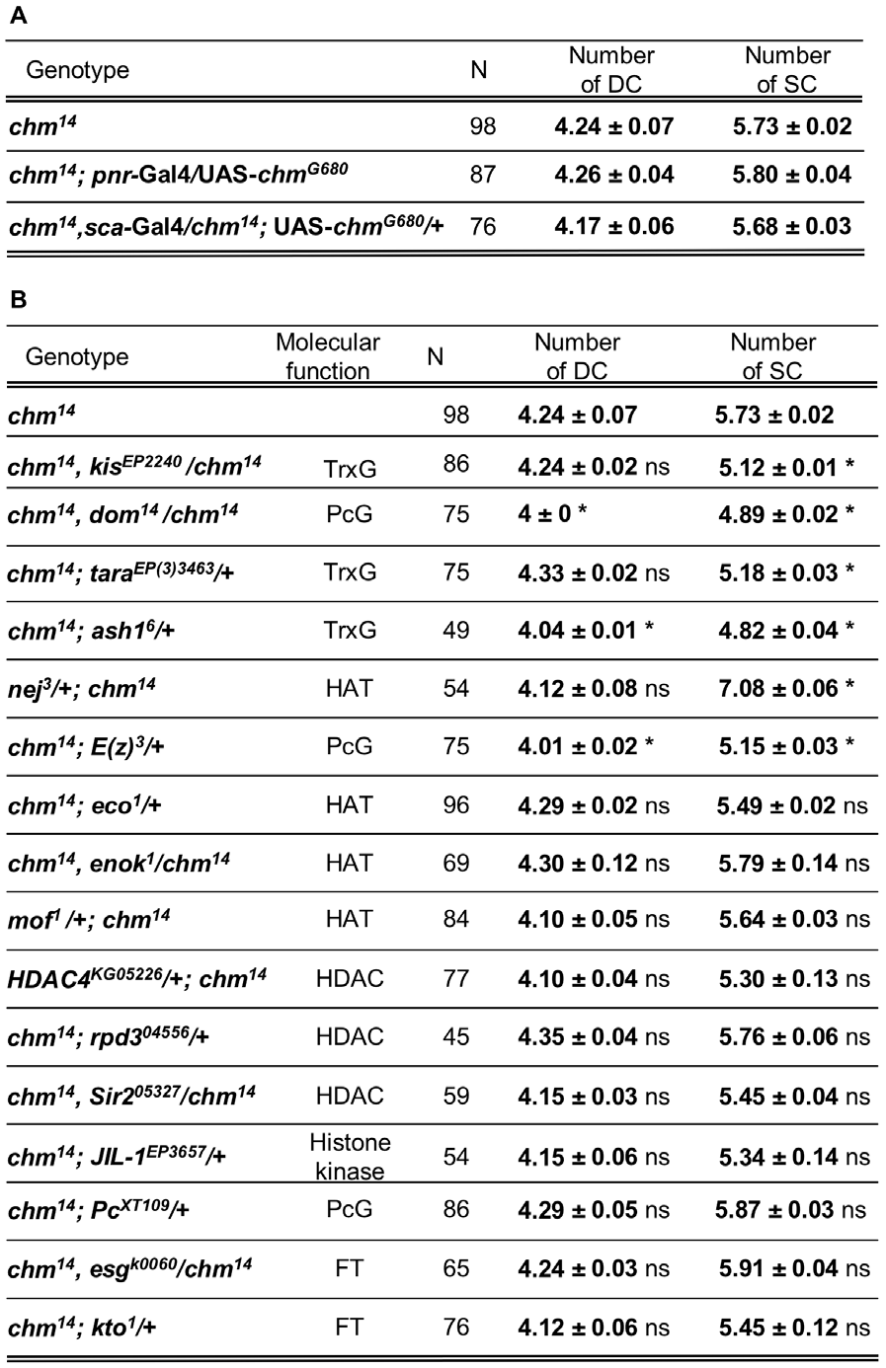
Chm epigenetically controls macrochaete formation. (A) Chm putative HAT activity is required for SOP determination: No rescue of *chm* extra bristle phenotype upon expression of the Chm^G680^ variant in DC and SC proneural clusters (*sca*-Gal4; *pnr*-Gal4). (B) Summary of genetic interactions between *chm* and 16 genes encoding chromatin remodeling factors. No change in DC or SC bristle number was observed in heterozygotes for each of the 16 genes, except for *ash1^6^/+* and *tara^EP(3)3463^/+* which exhibit 4.07 and 4.18 DC, respectively, and a normal number of 4 SC. Molecular function of each factor is given. Bristle scoring were compared using Student's *t* test: *, statistically different with regards to *chm* mutant flies, *P*<0.05; ns, not statistically different. N, number of thoraces examined.

Having established that Chm is an important regulator of SOP specification, we asked whether its putative acetyltransferase activity was required in this process. We used a Chm derivative (Chm^G680^) bearing a mutation at an invariant position of the acetyl-CoA binding site. A similar mutation has been reported to impair the enzymatic activity of MYST histone acetyl transferases (HATs; i.e. Sas3, Esa1 and Mof) [46]–[48] and we previously demonstrated that this mutation impairs Chm ability to specifically complement Sas2-mediated yeast telomeric position effect [30]. We thus tested the ability of Chm^G680^ [31], to rescue *chm* extra DC and SC bristle phenotypes. In contrast to what previously observed with wild-type Chm (Fig. 1B), providing the Chm^G680^ variant in the *pnr* expression domain (*pnr*-Gal4) or in the proneural clusters (*sca*-Gal4) does not allow rescuing *chm* bristle phenotypes (Fig. 4A). Since Chm and Chm^G680^ Myc-tagged constructs are expressed at similar levels [31], we concluded that Chm represses neural fate in DC and SC proneural clusters in an acetyltransferase activity-dependent manner.

Since chromatin structure regulation often requires a complex interplay between multiple chromatin remodeling complexes, we next investigated whether already-identified chromatin modifiers could be involved with Chm in the determination of SOPs in proneural clusters. Using the loss-of-function *chm* mutant as a sensitized background, we screened for mutations in genes encoding chromatin proteins (i.e. 16 genes tested) able to dominantly modify *chm* DC and SC bristle phenotypes. In addition to already-mentioned mutations in the TrxG members *osa* and *mor* (Fig. 3B), we found that mutations in *kismet* (*kis*), *domino* (*dom*), *taranis* (*tara*), *absent, small, or homeotic discs 1* (*ash1*), *nejire* (*nej*)/*CBP* and *Enhancer of Zeste* (*E(z)*) dominantly modify *chm* sensory bristle phenotypes (Fig. 4B). Among these genes, *dom*, *ash1* and *E(z)* affect both DC and SC phenotypes, while *kis*, *tara* and *nej*/*CBP* affect the SC bristle phenotype only. Of note, and validating our approach, several of these genes (*kis*, *tara* and *nej/CBP*) were found to play a role during SOP determination in the thoracic expression domain of Pnr [49].

## Discussion

### *chm* is required to restrict sensory organ formation

This study uncovers a novel role of Chm during adult thorax development. We report that, in addition to a defect in thoracic closure, homozygous null pharate adults exhibit supernumerary dorsal macrochaetes. Contrasting however with thoracic closure, where *chm* modulates JNK signaling during wing disc migration and fusion along the midline [31], its function during macrochaete development is genetically independent of JNK signaling activity (Table 1). Furthermore, the histone deacetylase Rpd3 counteracts Chm-mediated epigenetic control of JNK target genes during thorax closure, as mutating one copy of *rpd3* rescues the thoracic cleft of *chm* mutants [31]. Conversely, *rpd3* has been found here not to genetically interact with *chm* during sensory bristle formation (Fig. 4B). Thus, Chm is likely recruited in distinct molecular pathways during thorax closure and dorsal macrochaete specification.

*chm* mutants express a mild neurogenic phenotype, restricted to one supernumerary aSC and occasionally one aDC per hemithorax, emerging from the SC and DC proneural territories. Of note, restoring in a *chm* mutant background *chm* expression in proneural clusters (rescue by *sca*-Gal4 or *pnr*-Gal4) impairs the formation of extra but not of normal sensory bristles. Thus, Chm does not play an essential role in neural fate repression, but definitively acts to restrict neural fate commitment within DC and SC proneural clusters. Another feature of the phenotype lies in the systematic occurrence of epidermal cells between the ectopic and the normal macrochaete, indicating that Notch-mediated lateral inhibition is still operating, consistent with the fact that genetic alteration of the Notch-Delta regulatory loop does not modify the penetrance of *chm* bristle phenotype (Table 3). Likewise, we failed to detect any genetic interaction between *chm* and genes acting in Wg and Dpp signaling pathways (Fig. 3A) known to be involved in bristle patterning on the notum [50]. Altogether, these data suggest that Chm restricts neural fate in the DC and SC proneural clusters without influencing lateral inhibition and prepattern establishment.

Driven after SOPs are selected (*neur*-Gal4) in phenotypic rescue experiments, Chm is no longer able to restore appropriate macrochaete number, indicating that its function of neural fate restriction is required prior SOP singling out. In addition, the epistatic status of *ac-sc* to *chm* suggests that Chm may act upstream of *ac-sc* genes to repress ectopic bristle formation from SC and DC proneural clusters; that Chm may regulate Ac and Sc transcriptional activity to prevent extra-SOP emergence; or that Chm restricts SOP emergence through a mechanism yet to be described.

### An epigenetic network controls SOP determination

Increasing evidence involves chromatin dynamics as an important regulatory level during sensory organ development, and several chromatin factors or complexes have been proposed to function in the control of proneural gene expression [26], [28], [29], [51]. Here, we have shown that Chm is required within the DC and SC proneural clusters to restrict the neural fate to two SOPs only. Phenotypic rescue experiments furthermore revealed that Chm fulfills this function in a manner dependent of its putative HAT domain (Fig. 4A). Indeed, a mutation in the acetyl-CoA binding domain (Chm^G680^) prevents the rescue of *chm* extra DC and SC phenotypes.

HATs are often involved in defining landscapes of open chromatin that are permissive for transcription initiation [52]. Thus, Chm function as a negative regulator of transcription is counter-intuitive. In contrast, Chm may enhance the expression of a transcriptional repressor that would in turn repress the neural fate. Alternatively Chm may acetylate non-histone substrates to regulate their stability, their localization, their activity and eventually their function. Both Pannier and Scute are negatively regulated by phosphorylation in the wing epithelium [53]. Whether acetylation also controls their function should be tested.

Our candidate genetic screen identified several chromatin factor encoding genes that interact genetically with *chm*, either positively such as *mor* and *osa* TrxG members and the HAT encoding gene *nej/CBP*, or negatively such as three other TrxG genes, *kis*, *tara* and *ash1*, and two members of the PcG, *E(Z)* and *dom* (Fig. 4B). It is interesting to note that *chm* and TrxG/PcG genes act together in other developmental processes: *osa*, *tara* and *chm* have been recently simultaneously found in a suppressor screen of wing dorso-ventral boundary defect [34]; *chm* mutation enhances homeotic phenotypes of TrxG (unpublished) and of PcG genes as well [30], indicating a function in both PcG-mediated silencing and TrxG-mediated maintenance of Hox gene activity. Mor and Osa are TrxG subunits of the Brm complex, thus the enhancement of the *chm* bristle phenotype resulting from the removal of one copy of *mor* or *osa* genes is in agreement with Chm synergizing with the Brm complex to counteract the neural fate. Kis, Tara and Ash1 TrxG members do not belong to the Brm complex, and have not yet been assigned with a function in sensory organ formation. The dominant suppression of the bristle phenotype in *chm* animals heterozygous for the encoding genes suggests that Kis, Tara and Ash1 play a role opposite to that of Chm in the control of SOP specification, and act at a level different from the Brm complex to facilitate the neural fate. Recent studies shed new light on Kis/Ash1 and Brm complex interplay during transcriptional activation [54], [55]. The Brm remodelling complex is proposed to act in first for chromatin opening and coactivator recruitment, and the Kis-L protein to function afterwards, as a monomer required for the recruitment of the histone methyltransferases Ash1 and Trx that lay down a methylation mark promoting early steps of transcriptional elongation. Although this sequence has been described in contexts where the Brm complex acts as an epigenetic activator, a similar mechanism might also account for a putative repressive activity, assuming for instance that chromatin remodeling by the Brm complex allow corepressor recruitment. In this case, we speculate that Chm would favor this negative effect of the Brm complex on Pnr function and impair Kis/Ash1-mediated activation. Transcriptional repression by PcG proteins is also likely to take place during SOP determination, in a manner opposite to Chm, as suggested by negative genetic interactions of *chm* with *E(Z)* and *dom* (Fig. 4B), A role for PcG factors in proneural gene control is for instance supported by the observation that *Polycomb* mutation enhances the extra bristle *pnr^D1^* phenotype [28]. Finally, the synergistic effect of *chm* and *nej/CBP* mutations in extra bristle formation (Fig. 4B) supports further that the chromatin structure and histone tail acetylation play an important role in the neural vs epidermal fate decision. Further work will be necessary to approach at the molecular level whether and how Chm interferes with *achaete/scute* genes, transcription factor Pnr and TrxG/PcG factors in chromatin-mediated control of sensory organ development.

Chm is conserved in mammals, where it is known as HBO1, KAT7 or MYST2 [56]. Studies in human cancer cells have mostly focused on its function in DNA replication initiation and its involvement in inhibiton of growth (ING) protein-based complexes [57]–[60]. KAT7 behaves as a negative or positive regulator of gene expression. For instance, in gene reporter assays, it represses androgen receptor and NF-kappaB mediated transcription [61], [62] while it enhances AP-1 and p53 mediated transcription [63], [64]. KAT7 molecular role(s) in gene expression is still barely understood and therefore the direct or indirect effects of KAT7 in each study remain to be assesed. The phenotype of KAT7 knock-out mice was recently reported and KAT7 is essential for early mice embryogenesis [65]. In contrast to studies in human cancer cells, KAT7 appears dispensable for DNA replication in mice embryos while being required for gene regulation. Whether, as Chm in *Drosophila*, KAT7 also function in parntenrship with GATA factors to control neural develoment remains to be investigated.

### Hypothetic function of Chm in SOP determination

Several lines of evidence suggest that Chm may affect the function of Pnr. Firstly, mutants loss-of-function of *pnr* present similar phenotypes as Chm, including extra-SC and DC bristles. Secondly, *pnr* and *chm* genetically interact in a manner consistent with their products playing opposite roles during SOP cell fate commitment, since loss-of-function of *pnr* (*pnr^VX^* alleles) dominantly rescues *chm* bristle phenotypes, and *pnr^D1^*, commonly presented as a gain-of-function allele producing a constitutive activator unable to recruit the corepressor Ush, dominantly aggravates the extra DC *chm* phenotype (Table 2). The dominant rescue of the extra SC phenotype rather suggests that *pnr^D1^* conversely behaves as a loss-of-function allele in the SC cluster. In support of this, previous reports have proposed that the SC (although not molecularly identified) and DC enhancers display different topographies in binding sites for Pnr and for Pnr cofactors [23], [66], [67]. Thirdly, *chm* genetically interacts with four of the genes known to interfere with *pnr* in the transcriptional control of *ac-sc*: *ush*, *Chi*, *mor* and *osa*. Of particular interest is the dual function of *ush*, which synergizes or antagonizes *chm* activity during DC or SC macrochaete formation, respectively. These opposite effects are reminiscent of the genetic interactions discussed above between *chm* and *pnr^D1^*, supporting further that the partnership between Pnr and Ush may be different in the SC and DC cluster.

These genetic interactions, dominant phenotypic suppression by mutation in genes encoding Pnr coactivators (Ush in the SC cluster, Chi) and dominant phenotypic enhancement by mutation in genes encoding Pnr corepressors (Ush in the DC cluster, the Brm complex members Mor and Osa), are suggestive, yet not demonstrative, of a role for Chm in counteracting *ac-sc* activation by Pnr. Given the network of genetic interactions observed with chromatin regulators, this could be achieved by regulation of the higher order chromatin structure or in the spatial organization of the *ac-sc* locus, which harbors multiple regulatory elements scattered throughout ∼100 kilo-bases of the X chromosome [4]. Also consistent with a role connected to chromatin proteins, Chm may be part of a PcG/TrxG maintenance mechanism that prevents *ac* and *sc* re-expression once cells are committed to the epidermal fate. In support of this model, PcG/TrxG response elements have been described in the *ac-sc* locus and *chm* was first identified has a PcG/TrxG genetic modifier [30], [68].

However, our data do not exclude that Chm controls SOP emergence at a step different than transcription of the *ac-sc* genes. Alternative models for Chm function in SOP emergence are discussed below. First, Chm may be involved in the activation of neurogenic downstream targets of Ac and Sc [69]–[71]. Second, *chm* mutation may alter mechanisms involved in the elimination of cells still expressing high levels of Ac and Sc after SOP differentiation to prevent excess SOP determination. Yet to be described for macrochaete development, SOP-like cells, expressing high level of Ac and Sc but not selected as SOPs, are eliminated by programmed cell death during microchaete development [72]. Thus, extra-SOPs in *chm* mutants may originate from cells normally eliminated. Intriguingly, we previously observed that *chm* activity is necessary for programmed cell death following distortion of proximo-distal information in the wing imaginal disc [31]. Third, Chm may be required in the SOP to repress the neural fate of cells not adjacent to the SOP. In addition to lateral inhibition, it has been shown that SOP inhibits cells over several cellular diameters by extending long filopodia, and that these filopodia remain after SOP selection [73]–[75]. This later model seems less likely, since supernumerary SOPs in *chm* mutants appear after the completion of the asymmetric division of the extant SOPs and thus *chm* extra-SC and DC bristles should be rescue by expression of UAS-*chm* in ‘normal’ neuralized-positive cells. Finally, Chm may interfere with a recently-described Daughterless/Emc regulatory loop, which defines proneural territories independently of Ac and Sc [76].

To conclude, our work identifies a new player in the control of macrochaete development on the notum of Drosophila melanogaster and suggests that epigenetic mechanisms are important in this process. Future work will nevertheless be necessary to unravel the molecular mechanisms underlying the selection of DC and SC SOPs by Chm.

## Materials and Methods

### Fly lines and manipulations

Flies were grown at 22°C on a molasse-yeast-gelatin standard medium. *pnr*-Gal4, *pnr^D1^*, *pnr^VX1^*, *pnr^VX2^*, *pnr^VX6^* and *ush^VX22^* lines were kindly provided by P. Heitzler (Strasbourg, France); MZ980-Gal4 by E. Martin-Blanco (Barcelona, Spain); *hep^1^* and *hep^75^* by S. Noselli (Nice, France); *mof^1^* by A. Akhtar (Heidelberg, Germany). All other fly stocks were obtained from the *Drosophila* stock centers in Bloomington and Szeged. Canton flies were used as wild type.

### Cloning and generation of pUAS-Chm^WT^ and pUAS-Chm^G680E^

The UAS-T-Chm^WT^ transgene encodes a Chm protein tagged in its N-terminus with 6 Myc-tags. It was generated by sub-cloning a Myc-tagged Chm fusion in the pUAS-T plasmid. To generate the pUAS-T-Chm^G680E^ plasmid, an internal fragment encompassing the HAT domain was removed from pUAS-Chm^WT^ by digestion with restriction enzymes AgeI and XbaI and replaced by the same AgeI/XbaI fragment from the Chm^G680E^ variant. The Chm^G680E^ variant was generated by site-directed mutagenesis [31]. The resulting pUAS-T-Chm^G680E^ and pUAS-T-Chm^WT^ are the same plasmids but the point mutation in the invariant Glycine. Different transgenic flies were generated by random insertion of the transgene.

### Histochemistry

Wing imaginal discs of third instar larvae (or young white pupae) were dissected in ice-cold PBS, fixed 15 minutes in PIPES 0.1 M, EGTA 2 mM, MgSO4 1 mM and formaldehyde 3.7, extensively washed with PBS, then with PBS/Triton 0.3%, stained during 30 minutes at 37°C with X-Gal 0.2% in 10 mM NaPi, 150 mM NaCl, 1 mM MgCl2, 3.3 mM K3Fe(CN)6, 3.3 mM K4Fe(CN)2,3H2O, pH 7.0. Imaginal discs were then washed in PBS to stop the staining reaction and mounted in 100 mM Tris-HCl pH 7.5, Glycerol 80% for observation under an Axiophot Zeiss microscope.

### Phenotype analysis

Mutant chromosomes used for genetic interactions assays were maintained over balanced chromosomes labeled by GFP (chromosome II) or *Tb* or *Ser* dominant markers (chromosome III) to allow genotypic selection in the progeny. 32 to 133 pharate adults of each genotype were dissected out of pupal cages and thoracic morphogenetic defects were scored under stereomicroscope.

### Statistical analyses

Each experiment was conducted in triplicate but the *hep^75^; chm^14^* genotype for which pupae from different crosses were pooled to obtain over 30 individuals. Standard error of the mean is reported in the different figures and tables (± s.e.m.). To determine statistical significance between two data sets, the Student *t*-test was performed: ns, no statistical difference; *, statistically significant difference *P*<0.05.
